# Early Disassembly of Femoral Head and Neck Components After Total Hip Arthroplasty Revision

**DOI:** 10.7759/cureus.13177

**Published:** 2021-02-06

**Authors:** Mehmed Nuri Tutuncu, Bedri Karaismailoglu, Erdem Sahin, Turgut Nedim Karaismailoglu

**Affiliations:** 1 Orthopaedics and Traumatology, Kars Harakani State Hospital, Kars, TUR; 2 Orthopaedics and Traumatology, Istanbul University-Cerrahpasa, Istanbul, TUR; 3 Orthopaedics and Traumatology, Samsun Medical Park Hospital, Samsun, TUR

**Keywords:** femoral head-neck dissociation, modular, revision hip arthroplasty, manufacturing error

## Abstract

Modular femoral and acetabular components are frequently used in hip arthroplasty. Although the use of modular components offers many advantages, the increased number of components leads to a high risk of disassembly. Disassociation of the femoral head and neck is a rarely reported complication in the literature. This case report depicts a patient with non-traumatic early disassociation of the femoral head and neck components following total hip revision arthroplasty. Femoral head-neck disassembly in early postoperative period may occur due to manufacturing error or insufficient impaction. If sufficient impaction is thought to be achieved, manufacturing errors should be kept in mind as potential underlying reasons for femoral head-neck dissociation.

## Introduction

Total hip arthroplasty is a very crucial and successful operation that ensures restoration of the hip joint and its functions. Modular hip prosthesis designs have been used since the early 1970s and became increasingly popular in the 1980s and 1990s, eventually turning into the gold standard. Modular prostheses provide flexibility during surgery, such as the selection of head and neck length, adjustment of leg length, and soft-tissue balance. Moreover, modular design enables the surgeon to change the problematic components without removing the well-fixed components during the revision surgeries which substantially reduces surgery duration and morbidity. However, modularity and an increased number of components have created some novel complications. The disassociation between the femoral head and neck appears as a little-known complication in the literature and its frequency has been reported to be 1-2/1000 [1].

The majority of the head-neck disassociation cases have been reported to occur following a forceful attempt of closed reduction after a total hip arthroplasty dislocation. Besides, gross trunnion failure due to crevice corrosion has been identified as an important cause of head and neck dissociation in the late period [2]. Only one non-traumatic early femoral head-neck disassociation is reported in the literature, which had developed following a total hip arthroplasty with a constrained cup [3]. The patient also had poliomyelitis and the disassociation was attributed to muscular imbalance and constrained cup usage by the authors. This case report depicts a case with non-traumatic disassociation of femoral head and neck in the early postoperative period following a hip revision arthroplasty using a non-constrained cup.

## Case presentation

**Figure 1:**
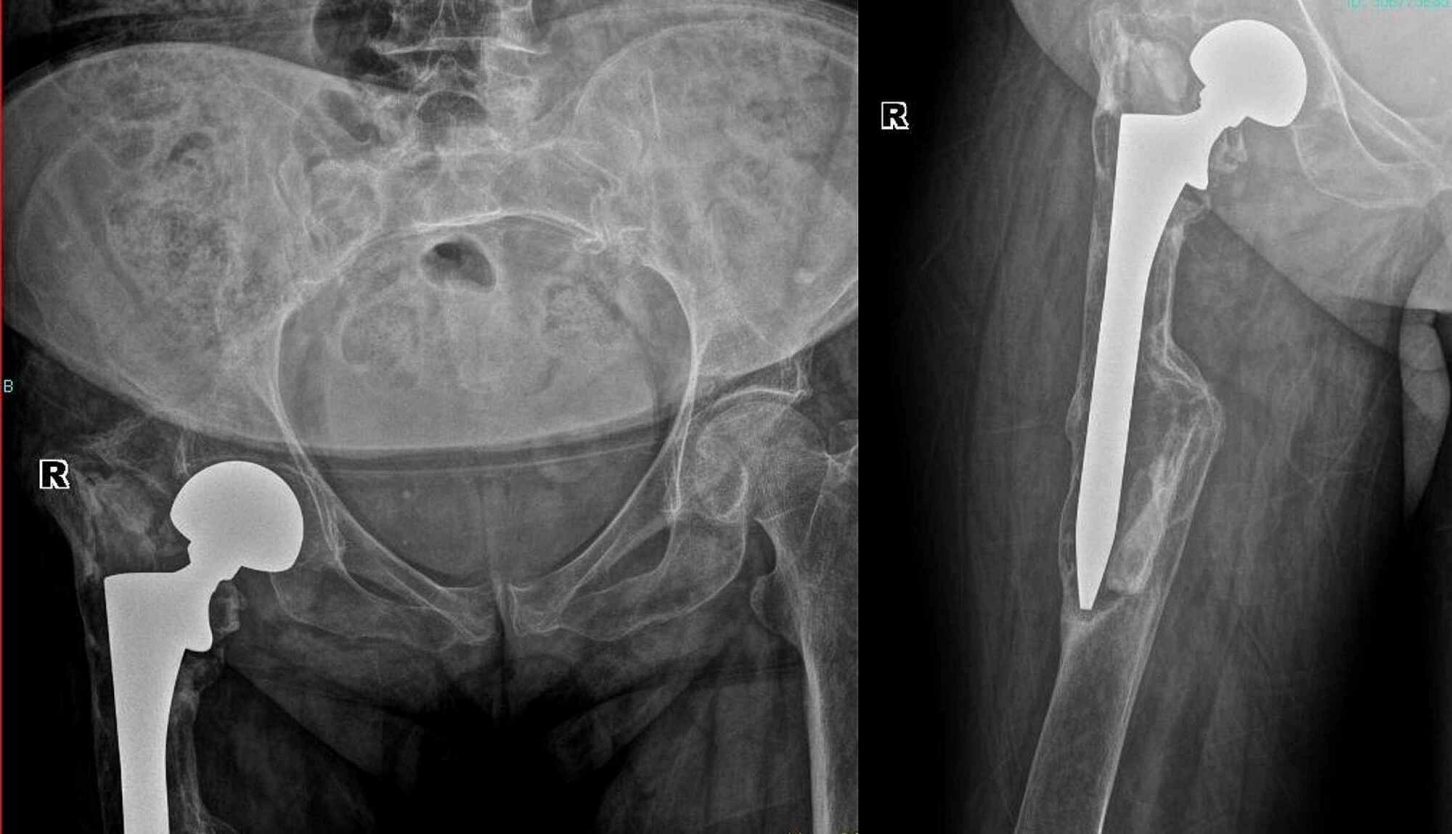
The preoperative radiographs of the patient with loosening of the right hip prosthesis.

A 76-year-old female patient who underwent bipolar hemiarthroplasty due to right femoral neck fracture six years ago, presented with increasing pain in her right hip. Her complaints had started approximately three years ago. Implant loosening and malposition were detected in the right hip prosthesis; therefore, revision surgery was indicated (Figure 1).

**Figure 2:**
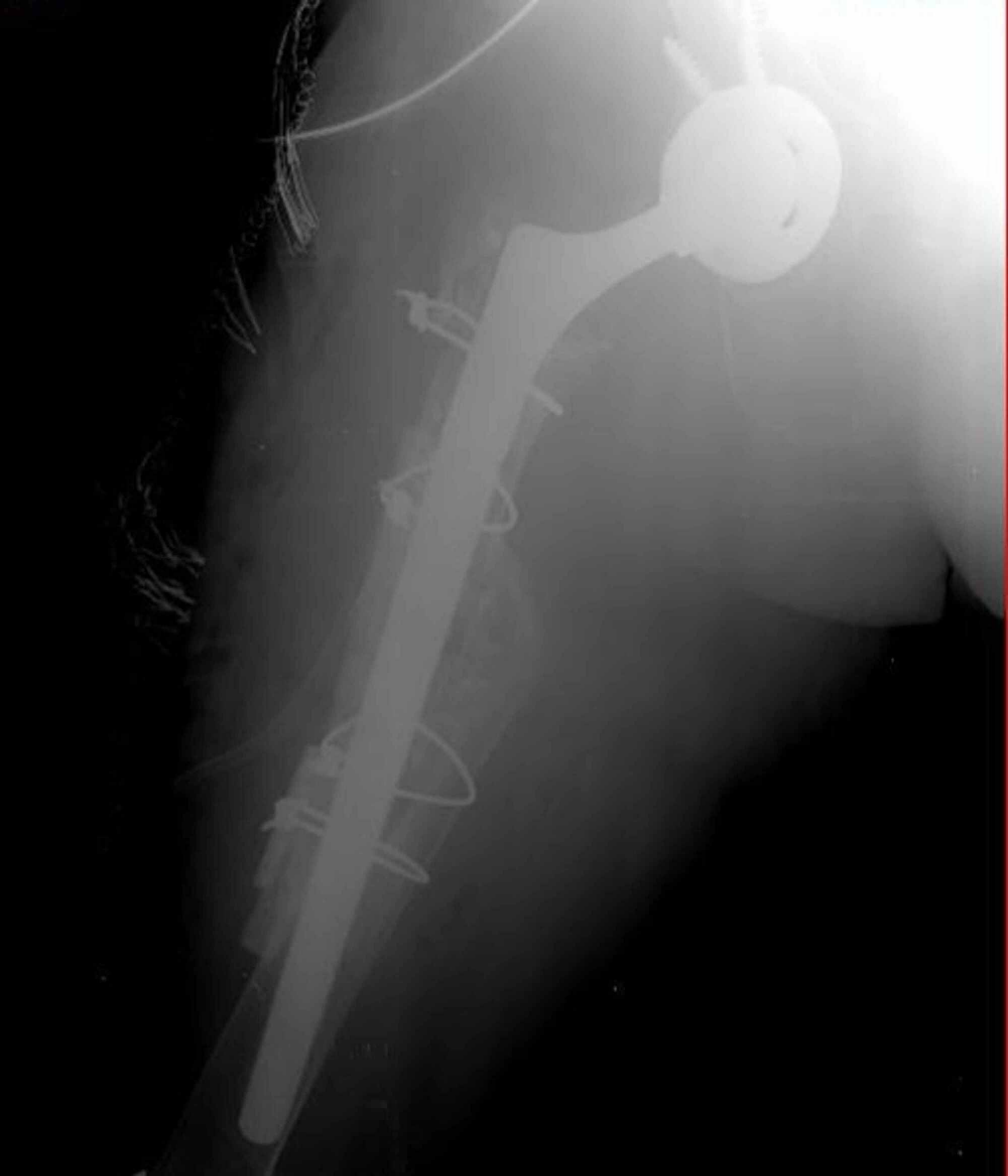
Early postoperative radiography of the patient who underwent revision total hip replacement.

The patient underwent hip arthroplasty revision surgery with a posterior approach (Figure 2).

Acetabular cup no. 48, CoCr femoral head no. 32 (+4 mm) and lateralized offset neck (+4 mm) (Lima Corporate, Italy) were used. The femoral head was impacted on the neck with an appropriate force and the hip was reduced.

**Figure 3:**
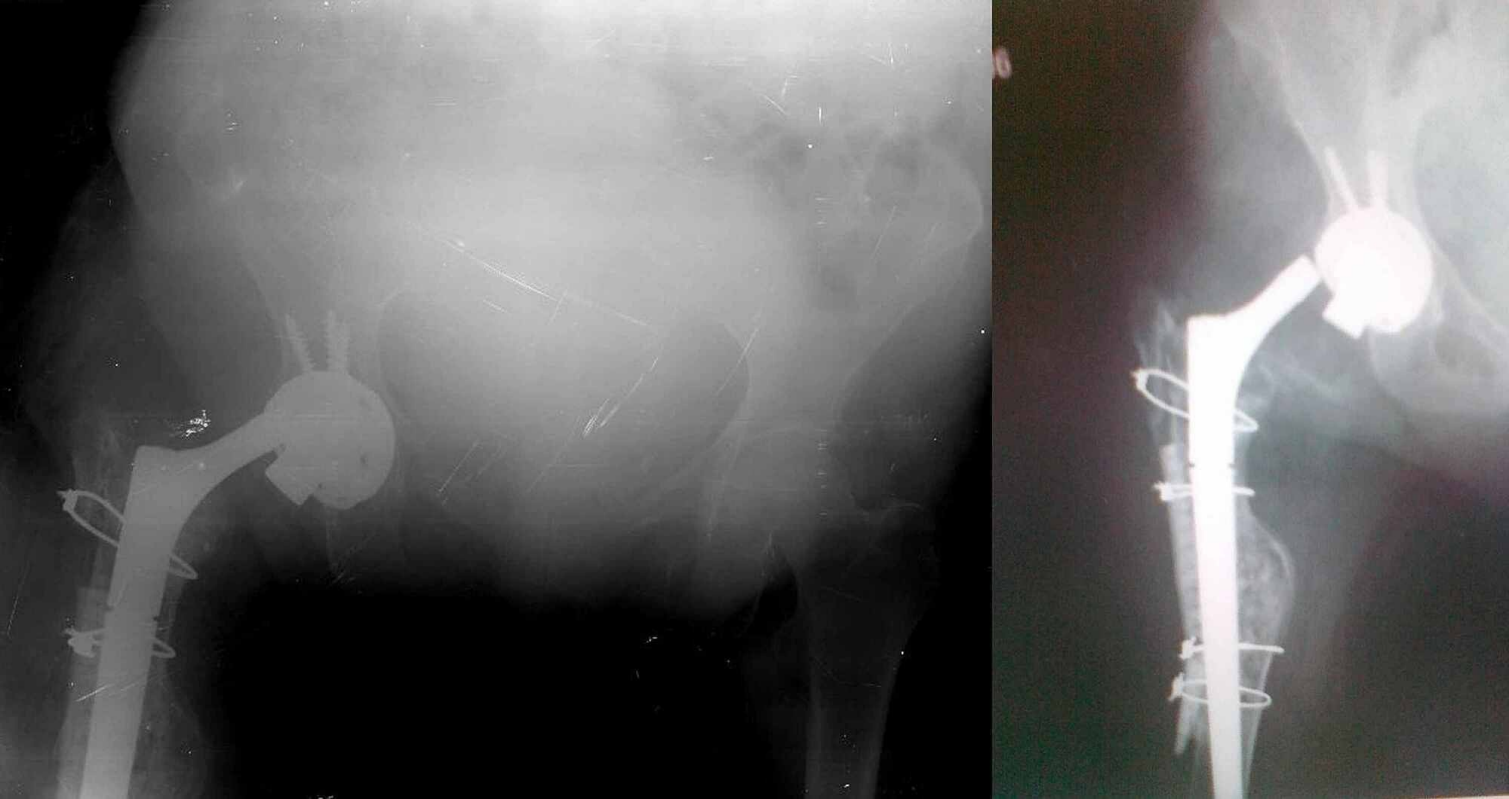
The direct radiographs of the patient three months after the surgery showing disassociation of the femoral head and neck components of the hip prosthesis.

Weight-bearing was allowed at the end of sixth week. At third-month follow-up, the patient was able to mobilize pain-free. However, a few days after her third-month follow-up, she was admitted to the outpatient clinic with complaints of severe pain and limitation of motion in the right hip without any history of obvious trauma. The patient reported that the pain and limitation emerged following a sound she heard while getting up from her seat. Disassociation of femoral head and neck components and accompanying hip dislocation was detected in the right hip (Figure 3).

The femoral head was in the acetabular cup. There were no signs of osteolysis or loosening of the components on the radiographs.

**Figure 4:**
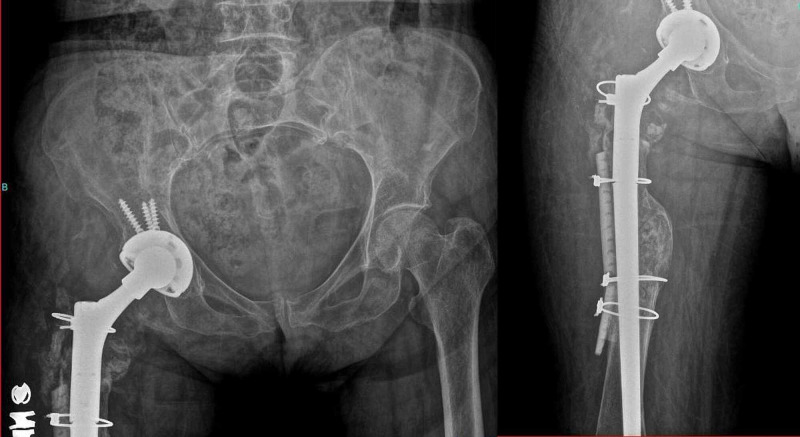
Postoperative one-year radiographs of the patient showing replaced femoral head, liner, and proximal modular part of femoral stem after revision due to the disassociation of the femoral head and neck components.

A same-sized femoral head, liner, and proximal modular part of femoral stem replacement were performed via the same anatomic approach (Figure 4).

Both distal part of the femoral stem and acetabular shell were in a good position as observed intraoperatively, thus were not revised. No apparent corrosion was detected on the removed components. There were no signs of metallosis in the surrounding tissues, nor any sign of an impingement that could potentially affect femoral head or neck. The retrieved parts of the prosthesis revealed that the head-neck interface was not matching appropriately although no visible damage was detected on the implants and the taper sizes (12/14) were appropriate for both neck and head components. The disassociation was attributed to a possible manufacturing error of either head or neck component. The hip joint was stable after revision. No problems were encountered during the patient’s three years of follow-up.

## Discussion

The greatest advantage of the designs with a modular femoral neck that connects with the modular femoral head and stem is the ability to independently adjust and optimize femoral anteversion, leg length and femoral component offset [4]. However, the increased number of modular junctions leads to more complications. The most common complications of modular hip prosthetic designs are corrosion and loosening of the component surfaces. The hip dislocation can also be encountered depending on the surgical approach, restoration of soft tissue balance, prosthetic design, and placement of the components [5].

Disassociation between components can occur during closed reduction attempts following dislocations [6]. Sufficient impaction between the femoral head and neck is achieved following a strong blow with a 0.5 kg hammer, and the load exerted during activities of daily living may also strengthen the impaction [7]. Non-traumatic dislocation in which the femoral head remained in the acetabular cup is rarely reported [8]. In late non-traumatic dissociations, the crevice corrosion phenomenon has been reported as the common cause [9]. Moreover, numerous risk factors, such as male gender, high activity level, BMI above 30, implant duration over six years, high offset, and femoral head above 36 mm, have been identified [10]. To the best of our knowledge, only one non-traumatic early femoral head-neck disassociation is reported in the literature which had developed following a total hip arthroplasty with a constrained cup [3], in which the disassociation was attributed to the force created by the constrained cup and also the patient had poliomyelitis leading muscular imbalance [3]. Our case is the first example of non-traumatic early femoral head and neck disassociation following a total hip arthroplasty revision, which does not involve a constrained liner. We think that the underlying reason was a manufacturing error preventing the correct matching and impaction. However, the limitation of this report is the lack of retrieval analysis to reveal the reason behind the failure.

## Conclusions

The orthopedic surgeons should always check if adequate impaction was achieved between the femoral head and neck by simply trying to remove the femoral head by hand before closing the incision. It should be kept in mind that head-neck interface can disassociate in case of a manufacturing error despite adequate impaction was applied.
